# Uncertainty Quantification in Hemodynamic Metrics from 4D Flow MRI with Super-resolution in a Carotid Bifurcation Model

**DOI:** 10.1007/s10278-025-01796-w

**Published:** 2026-01-07

**Authors:** Shaokai Zheng, Ali Mokhtari, Bernd Jung, Andreas Harloff, Dominik Obrist

**Affiliations:** 1 ARTORG Center for Biomedical Engineering Research, University of Bern, Freiburgstrasse 3, 3010 Bern, Switzerland; 2 Department of Diagnostic and Interventional Neuroradiology, Inselspital, Bern University Hospital, University of Bern, Bern, Switzerland; 3 Department of Neurology, Inselspital, Bern University Hospital, University of Bern, Bern, Switzerland; 4 Department of Diagnostic, Interventional and Pediatric Radiology, Inselspital, Bern University Hospital, University of Bern, Bern, Switzerland; 5 Translational Imaging Center, Swiss Institute for Translational and Entrepreneurial Medicine, Sitem-Insel, Bern, Switzerland; 6 Department of Neurology and Neurophysiology, Faculty of Medicine, Medical Center, University of Freiburg, Freiburg, Germany

**Keywords:** Carotid artery, 4D flow MR, Wall shear stress, Super-resolution, Computational fluid dynamics, Uncertainty

## Abstract

Super-resolution (SR) with supervised learning has emerged as a viable tool to enhance 4D flow magnetic resonance imaging (4DMR) data post hoc, but its impact on carotid hemodynamic measurements remains unclear. In this study, we quantified uncertainties in a phantom carotid artery model by comparing native 4DMR with and without SR against computational fluid dynamics (CFD) results. A phantom carotid artery model with steady flow was scanned at 3 Tesla. Magnitude images were segmented, and phase images were converted to velocities (3T). A pretrained 4DFlowNet was implemented to produce SR data (3T SR). A CFD simulation was performed on the same set up, serving as a reference. Uncertainties of geometrical processing were assessed by surface and Hausdorff distances. Hemodynamic metrics included intraluminal velocity and vorticity statistics, flow rate, and wall shear stress (WSS). A paired two-sided Wilcoxon signed-rank test was used to test the comparisons. Segmentations yielded a mean ± SD surface distance of 0.31 ± 0.06 mm (38 ± 7.5% of voxel size) and a Hausdorff distance of 1.2 mm. SR processing improved flow patterns and statistics of intraluminal hemodynamic parameters (*p* = 0.033). For flow rate, standard deviation decreased from 14.7% (3T) to 2.8% (3T SR), but mean values showed larger deviation after SR processing. Regarding WSS, qualitative improvements were observed, but quantitative comparisons did not show significant difference (*p* = 0.20). Our results suggest that SR processing stabilizes flow rate estimates and improves intraluminal hemodynamic statistics. But applying SR on data with inadequate quality may lead to larger errors. Further efforts are needed to improve generalizability.

**Supplementary Information:**

The online version contains supplementary material available at https://doi.org/10.1007/s10278-025-01796-w.

## Introduction

Approximately 3–6% of the global population over 70 years of age are affected by carotid atherosclerotic stenosis (CA), and the prevalence increases with age [1]. It accounts for one in five patients with stroke or transient ischemic attack [2], the former being the leading cause of adult disability and death. Given the overall neutral effect of revascularization observed in the Second European Carotid Surgery Trial (ECST-2) trial [3], identifying high-risk patients has become increasingly important, at least for patients with a low to intermediate predicted risk of stroke.

Traditional risk assessment often relies on geometrical features, especially stenosis degree, as established in the North American Symptomatic Carotid Endarterectomy Trial (NASCET) [4] and the first ECST trial [5]. Later, with the advancement of imaging, the focus of risk stratification has shifted from stenosis quantification [5, 6] to plaque characterization [7, 8]. Features identifiable from plaque-magnetic resonance imaging (MRI), such as fibrous cap ulceration, lipid-rich necrotic core, and intra-plaque hemorrhage, have emerged as strong predictors of thrombo-embolic events, independent of the degree of stenosis [8, 9].

More recently, with the advancement of 4D-flow MRI [10], flow-derived features such as wall shear stress (WSS) along with its derived forms have been shown to correlate with plaque vulnerability and progression [11–13]. It is becoming a clinical routine in modern neurological clinics to screen patients with suspected CA. The associated hemodynamic analysis provides functional insights for patient triage, complementing structural imaging, and has attracted growing interest in computational and imaging-based hemodynamic studies.

However, the diagnostic power of WSS measurements depends on the accuracy of near wall velocity measurements and the delineation of vessel walls. Both are challenging tasks in the clinical setting. As a result, clinical evidence for the efficacy of WSS remains low, and it is not yet considered in the current guidelines for patient management [14, 15].

To address that, one possible solution is to enhance the 4D-flow MRI results by means of super-resolution (SR) [16] via supervised learning. The publicly available 4DFlowNet [17] has demonstrated reasonable improvement over native 4D-flow MRI results in cardiac and cerebral hemodynamic analyses [18–20]. Although it has not been applied in studies of CA patients or benchtop counterparts.

In this study, we aim to investigate the uncertainty of 4D-flow MRI measurements in the carotid artery with and without SR reconstruction as compared to the digital twin. For that, we performed an in vitro 4D-flow MRI measurement with clinical sequence on a phantom carotid model and repeated the same experiment in silico using computational fluid dynamics (CFD) simulation. Comparisons were made qualitatively and quantitatively on flow statistics, flow rate (*Q*), and wall shear stress (WSS). We anticipate that these results will provide useful reference data for incorporating 4D-flow MRI–derived hemodynamic features into digital models for carotid artery patient management.

## Materials and Methods

### In Vitro Experiment

**Fig. 1:**
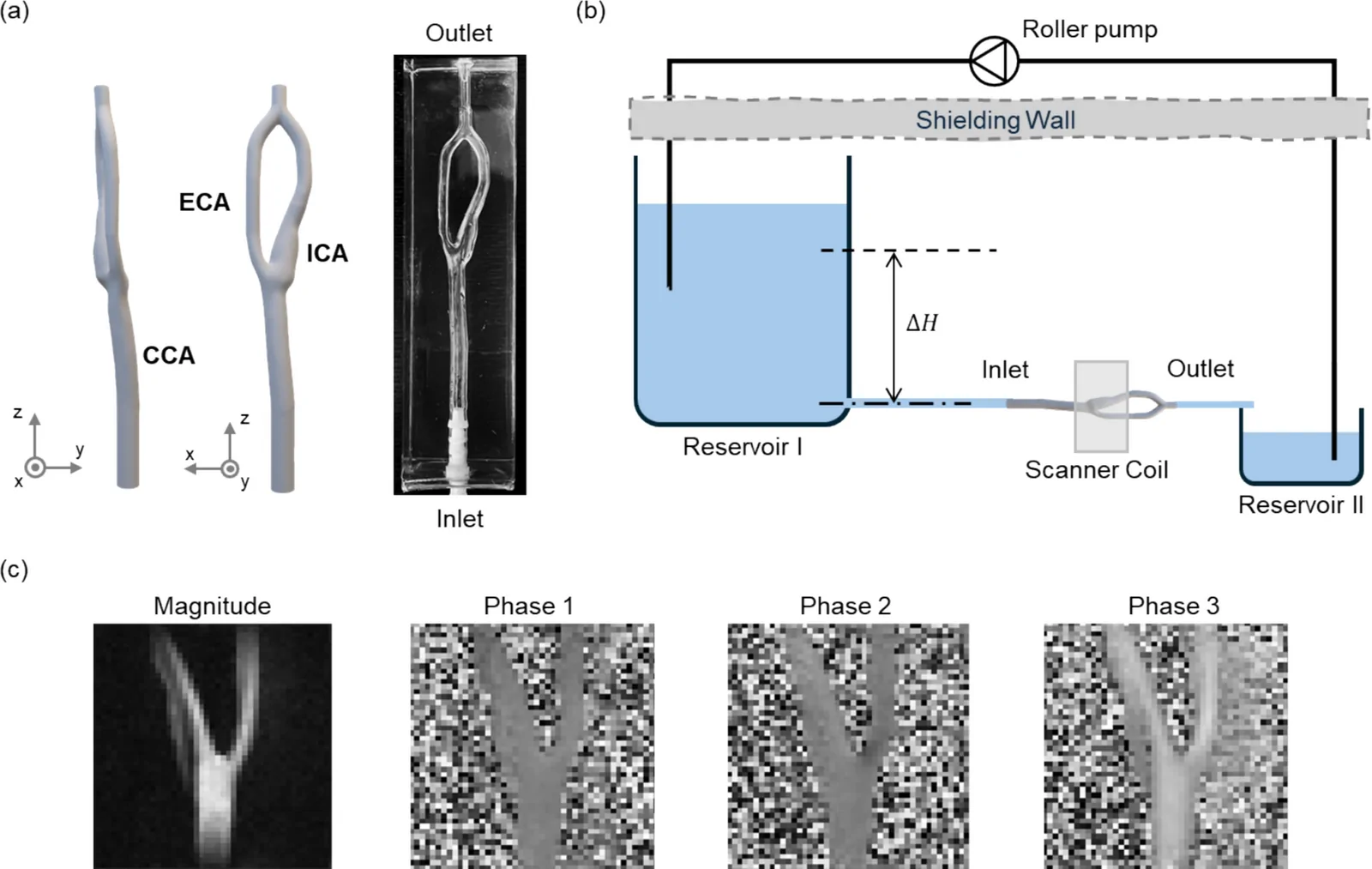
**a** The side view (left) and front view (middle) of the carotid model and its negative silicone phantom. **b** Illustration of the flow loop. **c** Sample magnitude and phase images from one time frame, cropped to region of interest

A carotid geometry from a healthy human subject (Fig. 1a) was taken from previously published studies [21], where the internal (ICA) and external (ECA) carotid arteries were extended and joined together to eliminate the ambiguity of having two outlet boundary conditions. A copy of the model is made publicly available at https://grabcad.com/library/carotid-bifurcation-with-joined-outlets-1. Further details on the phantom model are given in [22]. Briefly, a negative silicone phantom was manufactured by a commercial company with 3D-printed lumen mold and acetone bath. The silicone model was connected to a pressure-driven flow loop filled with deionized water (Fig. 1b), where the pressure was regulated by a pressure head ΔH and maintained by a roller pump placed outside of the scanning room. A steady flow rate of ${Q}_{ref}=1.7\text{ ml}/\mathrm{s}$ was established and was calibrated before data acquisition. For that, the weight of the water discharged from the outlet was measured at 90-s intervals and a linear regression was performed to calculate the actual flow rate. The flow was adjusted until the calculated flow rate based on the last four data points was within 1% of ${Q}_{ref}$. The steady flow rate was chosen to avoid the uncertainties caused by the compliance of the long tubing in case of a pulsatile flow.

The three-component velocity vectors were measured using a 4D-flow MRI sequence [23] with a 3 Tesla scanner (MAGNETOM Prisma, Siemens, Germany) from the Department of Neurology and Neurophysiology at the Freiburg University Hospital. Seventeen time frames were acquired using an artificial electrocardiogram-trigger signal with a cycle length of 1 s. Since the flow was steady, these time frames were treated as instantaneous measurements of the mean flow, which allowed us to look at the uncertainties of the measurement. For each time frame, one magnitude and three phase images were exported for further processing. The images were reconstructed with corrections to the gradient field inhomogeneities and noise. A bottle of water was placed next to the phantom for the eddy current correction similar to that described in [22]. Sample images from a time frame are given in Fig. 1c. Scanning parameters are given in Table 1.

**Table 1:** MRI acquisition parameters

Scanner model	MAGNETOM Prisma
Field strength [Tesla]	3.0
Echo time [ms]	4.5
Repetition time [ms]	7.2
Scan time [min]	~ 15
Flip angle [°]	12.0
Acquisition orientation	Coronal
Frequency-encoding direction	Head-foot
Acceleration (factor)	k-t GRAPPA (5)
VENC [cm/s]	22
Pixel spacing [mm]	[0.85, 0.85]
Slice thickness [mm]	0.8
Matrix size [pixel]	[224, 168, 48]

*GRAPPA* GeneRalized Autocalibrating Partially Parallel Acquisitions

### In Silico Experiment

First, the carotid model was discretized with tetrahedral elements, including three boundary layer elements with a stretching factor of 1.2, resulting in 7.3 million cells. The maximum cell size was 0.2 mm, which was 1/17 of the narrowest diameter of the geometry. The inlet at common carotid artery (CCA) branch was prescribed with a steady flow rate of ${Q}_{ref}=1.7 \mathrm{ml}/\mathrm{s}$ with parabolic velocity profile [24]. The outlet was prescribed with zero pressure with backflow suppression.

The Navier–Stokes equations were solved using the finite element software COMSOL Multiphysics® (V6.1) with the laminar flow model, which is appropriate for the flow conditions typically observed in carotid arteries. Steady-state solutions were computed with a Newtonian fluid model with density $\rho =998 \mathrm{kg}/{\mathrm{m}}^{3}$ and dynamic viscosity $\mu =1.002\times {10}^{-3}\text{ Pa}\cdot \mathrm{s}$, assumptions shown to yield reasonable results in simulations of carotid arteries [25]. The multifrontal massively parallel sparse direct solver (MUMPS) in COMSOL was used to solve the system with a relative tolerance of ${10}^{-8}$. The resulting velocity vectors with spatial coordinates were exported for further processing.

### Data Processing

For the 4D-flow MRI data, the magnitude image was used to segment the carotid geometry using a random walker segmentation method [26], and the surface was reconstructed with a marching cube algorithm (scikit-image v0.19.3) followed by Taubin smoothing (pyvista v0.45.2) on the surface. This was done for each time frame of the magnitude images, and the mean and standard deviation (SD) of the surface distance to the ground truth carotid model was calculated. The Hausdorff distance was calculated using the Euclidean distance (SciPy v1.13.1) between the surfaces.

**Fig. 2:**
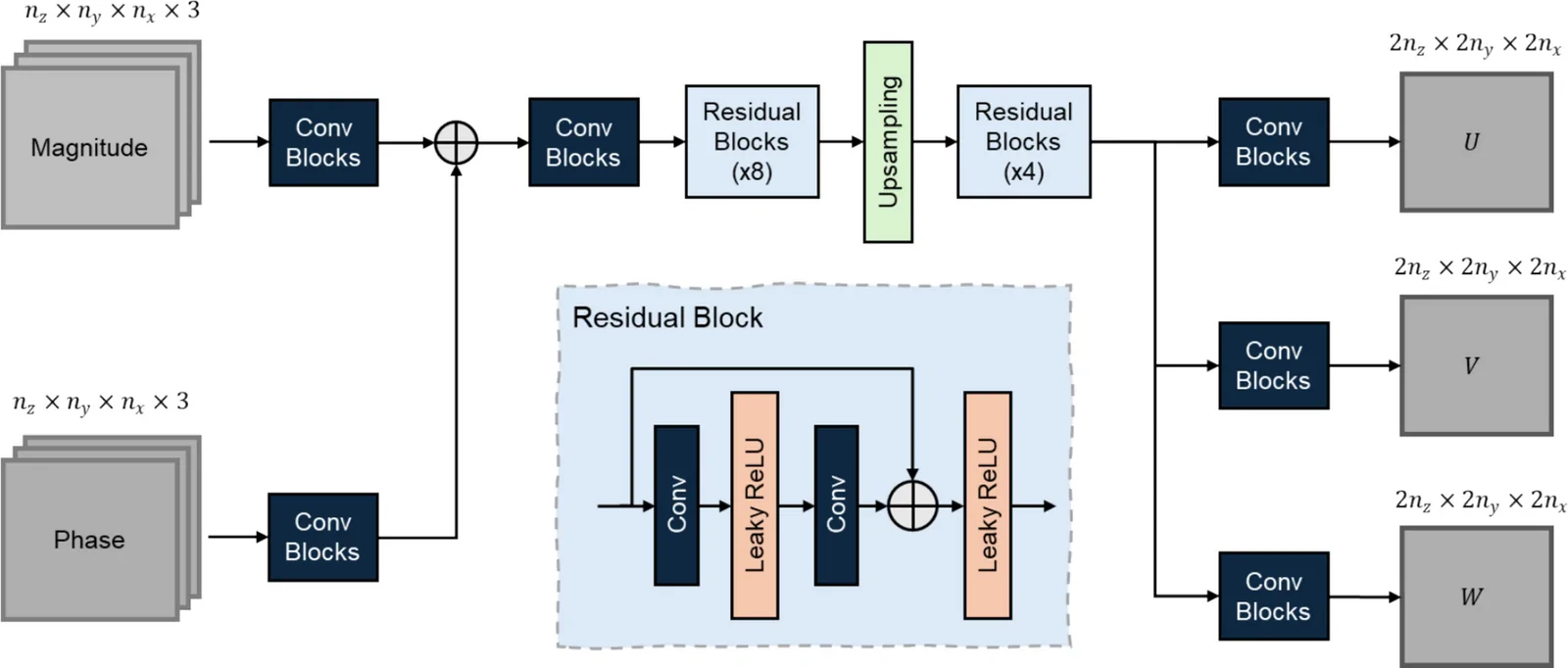
Architecture of the 4DFlowNet, repeated after [17]. The magnitude image, speed, and their product were used as input for the anatomical path

The velocity encoding (VENC) number was used to convert the phase images into velocity fields, referred to as 3T. Then, the pretrained 4DFlowNet-cerebral [17] model was applied to reconstruct the flow field, increasing the spatial resolution by a factor of  2. The super-resolved flow field is referred to as 3T SR. The architecture of the model is given in Fig. 2. The magnitude image, speed, and their product were used as input for the anatomical path of the network as defined in the original paper [18].

The training was done on four patient-specific CFD datasets of the cerebrovascular system, including the carotid arteries and the circle of Willis, with and without carotid stenosis [17]. Out of the four subjects, one presented no carotid stenosis, one presented with ipsilateral severe stenosis in the proximal ICA, and two were from the same patient with bilateral carotid stenosis before and after surgical re-opening of the proximal ICA. For each subject, low resolution MR images were synthesized by downsampling the CFD results onto a Cartesian grid with varying voxel sizes. Patches of the synthesized data were extracted and augmented with rigid rotations to boost the training sample size. The loss function was the sum of the voxel-wise mean squared error loss in the fluid and solid domains, respectively, in addition to an L2 regularization term. Further details of the training setup can be found in [17]. The model along with pretrained weights was implemented with TensorFlow (v2.10.0).

After acquiring the 3T and reconstructing the 3T SR velocity fields, contours of velocity magnitude were compared to CFD results at various *z*-planes to qualitatively assess the flow field. The velocity and vorticity fields of all time frames were averaged to obtain the mean field, and higher order statistics, i.e., skewness and kurtosis, were calculated to offer more information on the distribution of the variables. The WSS was calculated by fitting a polynomial function on the velocities sampled at one and two grid sizes inward along the wall normal direction, which proved to be a robust post-hoc method in previous studies [22, 27]. This was repeated for the CFD, 3T, and 3T SR data.

### Statistics

Quantitative results are reported as mean ± SD or median with interquartile range (IQR) as appropriate. To compare measurements, the mean absolute deviation (MAD) is calculated as:

$$\mathrm{MAD}=\frac1N{\textstyle\sum_{i=1}^N}\left|x-x_{ref}\right|$$

Where N is the total number of data, x is the measured value, ${x}_{ref}$ is the reference value, and $\left|\bullet \right|$ means the absolute value. Statistical measures of velocity and vorticity magnitudes were compared to quantify the relative error (RE), defined as:

$$\mathrm{RE}=\left|\frac{x - {x}_{cfd}}{{x}_{cfd}}\right|\times 100\mathrm{\%}$$

Where x is the measured hemodynamic parameter and ${x}_{cfd}$ denotes its CFD counterpart. Luminal flow rate (*Q*) was obtained by integrating the velocity through each *z*-plane, and the temporal mean and SD were calculated.

The nonparametric two-sided Wilcoxon signed-rank test is used to test the null hypothesis that the median difference between paired data is zero. A *p*-value below 0.05 is considered statistically significant. All statistical analyses were performed using the SciPy package (v1.10.1).

## Results

**Fig. 3:**
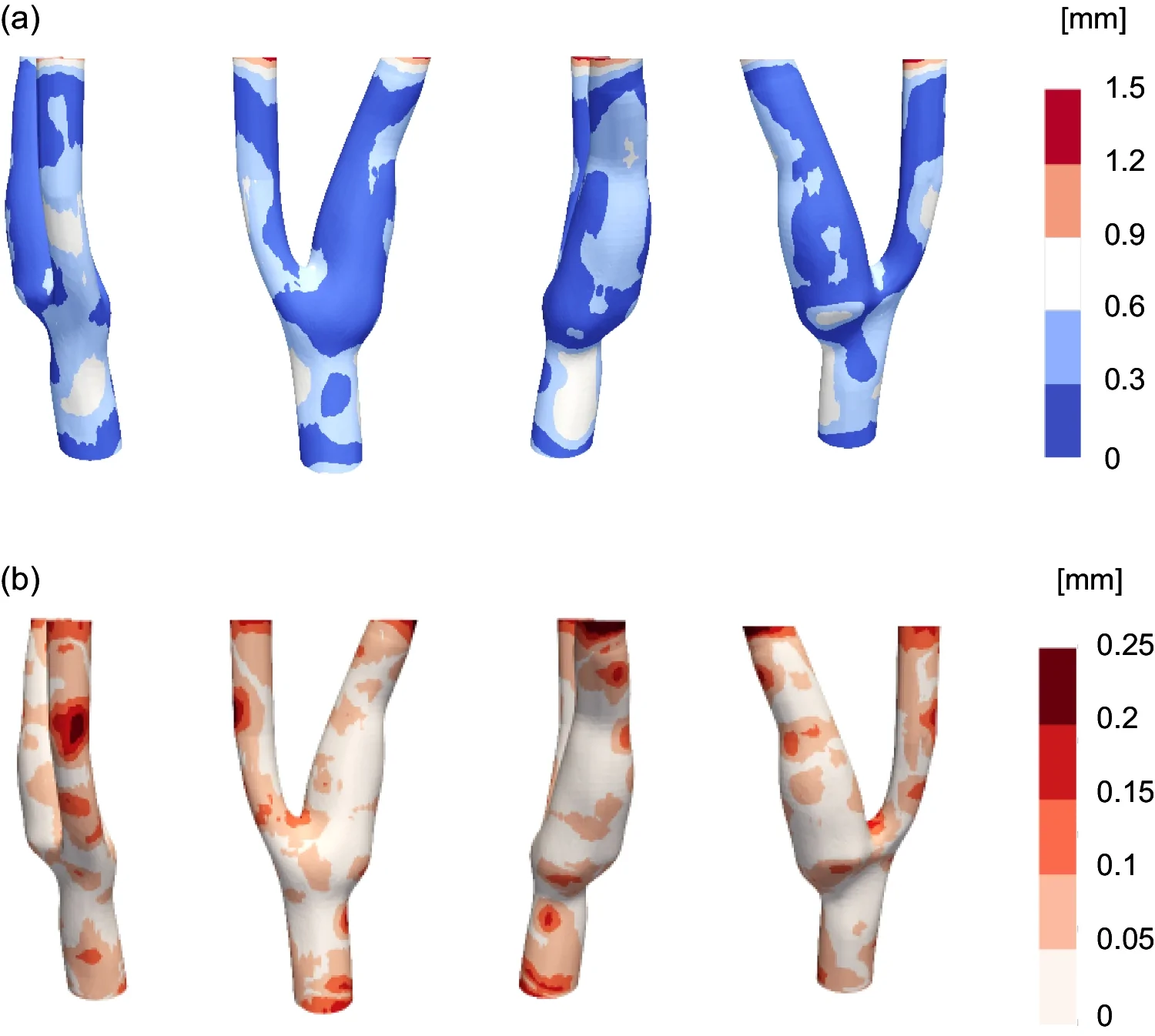
Geometrical processing error as (**a)** mean and (**b)** SD distance to the original surface. Four angles are presented by rotating the geometry along the longitudinal (z) direction

For geometrical processing, the temporal mean and SD distance between the reconstructed surfaces to the reference geometry are given in Fig. 3. Larger differences are observed close to the ICA/ECA end and in the middle of the ECA, where vessel diameter is the smallest (2–4 voxels). The spatial mean of the mean (± SD) distance was 0.31 (± 0.06) mm, equivalent to approximately 38% (± 7.5%) of the voxel size. The Hausdorff distance was 1.2 mm.

**Fig. 4:**
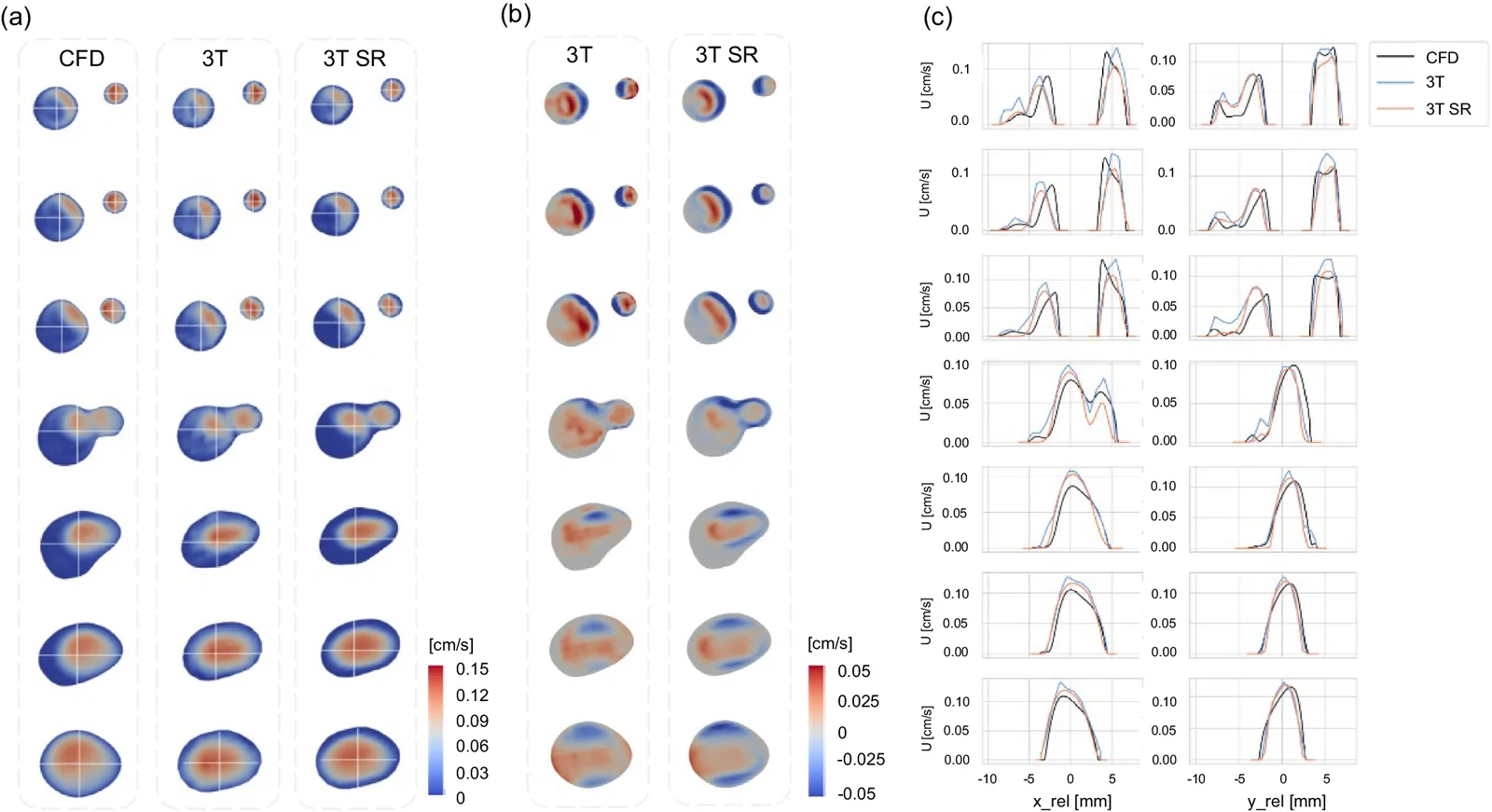
**a** Contours of the velocity magnitude on cross-sectional planes at different *z* locations for the CFD data, raw MR data (3T) and their super-resolved counterparts (3T SR). White lines crossing at the center of mass are used to extract velocities along *x* and *y* directions, respectively. **b** Contours showing the difference in velocity magnitude between 3T and 3T SR against CFD results, respectively. **c** Extracted velocity profiles at each plane. Coordinates x_rel and y_rel are relative distances from the center of mass. Profiles from ICA (ECA) are shifted by − 5 (+ 5) mm for better visibility

The cross-sectional planes of velocity magnitude contours along the longitudinal (*z*) direction are given in Fig. 4a, demonstrating the enhanced flow patterns after SR reconstruction. From each plane, the difference in velocity magnitude between 3T and 3T SR against CFD results is presented in 2D contours (Fig. 4b). Velocity profiles extracted crossing the center of mass along the *x* and *y* directions are given in Fig. 4c.

Statistical measures including mean, SD, skewness, and kurtosis of intravascular parameters (velocity and vorticity magnitude) are given in Table 2 for three selected *z*-planes in the CCA, bifurcation, and ICA/ECA. For the three planes shown, the REs for 3T and 3T SR are not significantly different (*p* = 0.089). However, the RE for 3T SR is significantly smaller than that of the 3T case (*p* = 0.033) when more slices are included (see Table S1 in the Supplementary Material).

**Table 2:** Statistical measures at different z locations for 3T and 3T SR cases against CFD, along with the absolute error for each variable. The case with less relative error (RE) is highlighted in bold for each comparison

Location	Var	CFD	3T	3T SR	RE (3T)	RE (3T SR)
ICA/ECA (*z* = 66.30 mm)	Kurtosis velocity	− 0.476	0.032	− 0.629	1.07	**0.32**
	Kurtosis vorticity	2.027	2.836	1.867	0.40	**0.08**
	Mean velocity [m/s]	0.044	0.048	0.040	**0.09**	0.10
	Mean vorticity [1/s]	0.064	0.030	0.031	0.53	**0.51**
	SD velocity [m/s]	0.034	0.031	0.031	0.10	**0.10**
	SD vorticity [1/s]	0.055	0.018	0.025	0.68	**0.55**
	Skewness velocity	0.794	0.798	0.672	**0.01**	0.15
	Skewness vorticity	1.544	1.650	1.548	0.07	**0.00**
Bifurcation (*z* = 57.03 mm)	Kurtosis velocity	− 0.549	− 0.592	− 0.885	**0.08**	0.61
	Kurtosis vorticity	− 0.284	− 0.700	− 0.251	1.46	**0.12**
	Mean velocity [m/s]	0.031	0.040	0.033	0.28	**0.08**
	Mean vorticity [1/s]	0.030	0.029	0.026	**0.04**	0.13
	SD velocity [m/s]	0.034	0.034	0.040	**0.01**	0.17
	SD vorticity [1/s]	0.025	0.016	0.027	0.37	**0.06**
	Skewness velocity	0.940	0.797	0.831	0.15	**0.12**
	Skewness vorticity	0.730	0.444	0.949	0.39	**0.30**
CCA (*z* = 52.4 mm)	Kurtosis velocity	− 1.118	− 1.224	− 1.439	**0.09**	0.29
	Kurtosis vorticity	− 0.524	− 0.729	− 0.703	0.39	**0.34**
	Mean velocity [m/s]	0.063	0.061	0.059	**0.04**	0.07
	Mean vorticity [1/s]	0.058	0.036	0.039	0.38	**0.32**
	SD velocity [m/s]	0.034	0.036	0.040	**0.05**	0.18
	SD vorticity [1/s]	0.034	0.017	0.024	0.50	**0.27**
	Skewness velocity	− 0.169	0.147	0.007	1.87	**1.04**
	Skewness vorticity	0.596	0.330	0.648	0.45	**0.09**

**Fig. 5:**
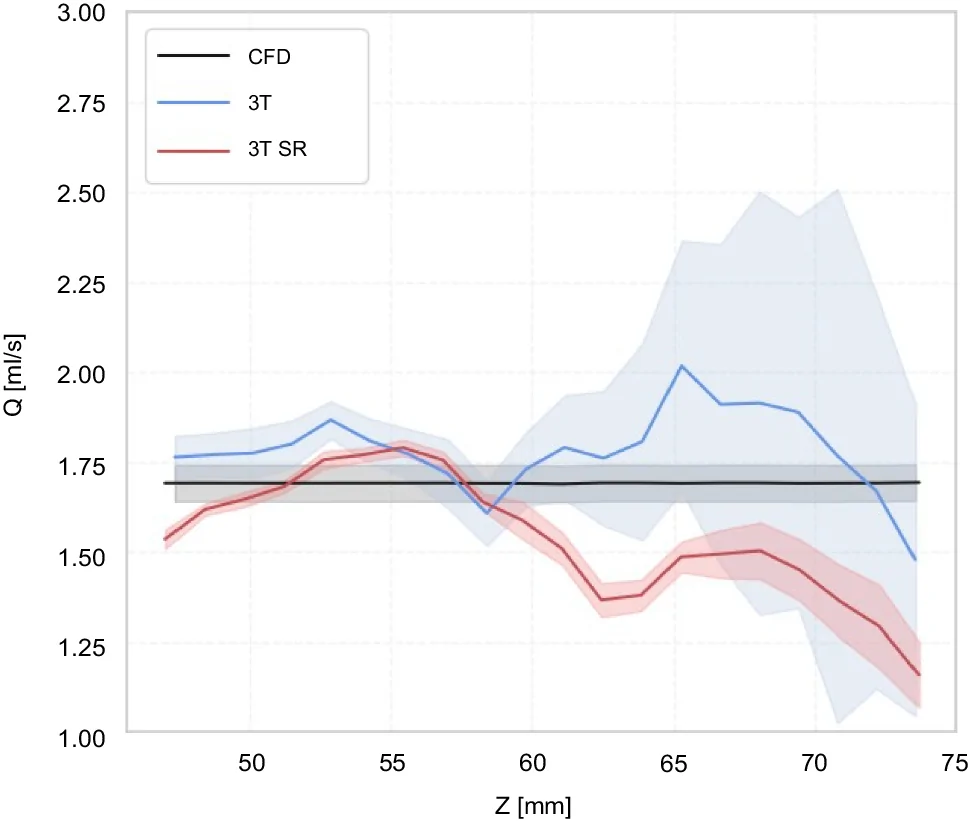
Flow rate (*Q*) along the longitudinal (*z*) direction as calculated from the CFD (black), native resolution MR (blue), and the super-resolved data (red) ± SD (shaded area). For CFD data, a ± 3% SD is manually added

Regarding flow rate *Q*, the 3T SR reconstruction demonstrated much less variance (Fig. 5), especially in the ICA/ECA branches beyond the apex of the bifurcation (*z* > 60 mm). The mean flow rates, however, were consistently underestimated in the ICA/ECA branches. The MAD of *Q* (as percentage of ${Q}_{ref}$) was 0.12 ml/s (6.8%) and 0.19 ml/s (11.1%) for the 3T and 3T SR cases, respectively. The mean SD of Q was 0.25 ml/s (14.7%) and 0.048 ml/s (2.8%), respectively, for the 3T and 3T SR cases. If only the CCA section was considered, the MAD(Q) was 0.079 ml/s (4.7%) and 0.076 ml/s (4.5%), respectively, for the 3T and 3T SR case. The mean SD of Q in this case was 0.073 ml/s (4.3%) and 0.025 ml/s (1.4%) for the 3T and 3T SR case, respectively.

**Fig. 6:**
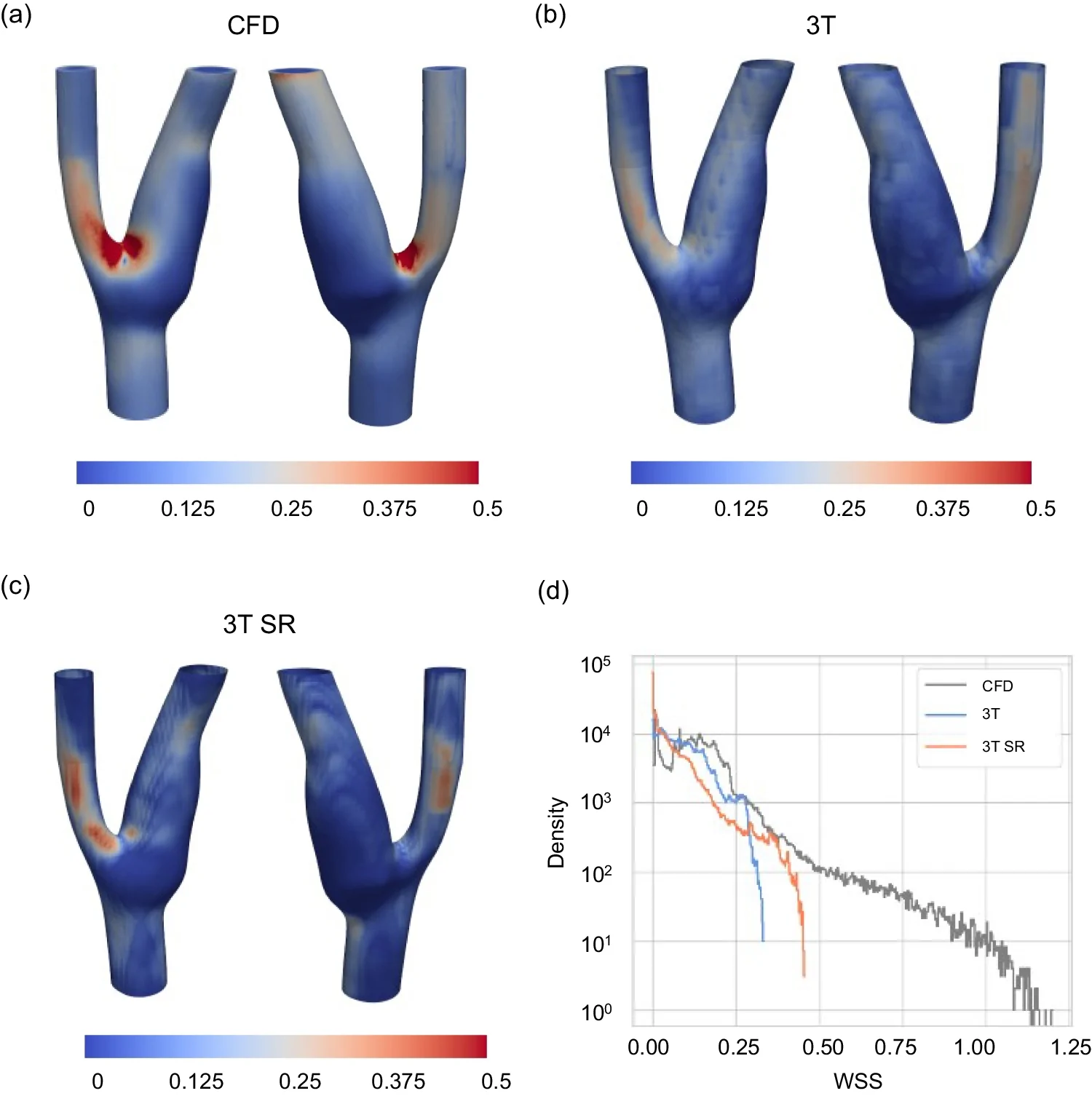
WSS from CFD (**a**), native 3T data (**b**), and 3T SR data (**c**) as surface contours and density distributions (**d**)

The WSS contours for the CFD, 3T, and 3T SR cases are given in Fig. 6. Qualitatively, both 3T and 3T SR cases underestimated the high WSS regions, especially around the apex of the carotid bifurcation. Although 3T SR cases demonstrated higher WSS in the ECA branch, especially on the ECA side of the apex of the bifurcation (Fig. 6c), the WSS patterns at the carotid bulb are better replicated by the 3T SR case too, supported by the better homogeneity of the low WSS region. The density distribution shows a higher peak WSS in the 3T SR case than the 3T case (Fig. 6d), although both 3T and 3T SR cases miss the higher WSS contents. Further statistics are given in Table 3. The REs for 3T and 3T SR cases, however, are not significantly different (*p* = 0.2).

**Table 3:** Statistics of WSS from CFD, 3T, and 3T SR results. The smaller relative error (RE) is highlighted in bold for each measure

	CFD	3T	3T SR	RE (3T)	RE (3T SR)
Maximum	1.2	0.33	0.46	0.73	**0.62**
Mean	0.13	0.09	0.066	**0.31**	0.49
SD	0.11	0.067	0.076	0.39	**0.31**
Quantile (97.5%)	0.4	0.26	0.3	0.35	**0.25**
Median	0.12	0.078	0.04	**0.35**	0.67
Quantile (2.5%)	0.0055	0.0024	0.0002	**0.56**	0.96
IQR (75–25%)	0.11	0.098	0.084	**0.11**	0.24

## Discussion

In this study, the uncertainties for geometrical and hemodynamic processing of 4D-flow MRI data were investigated using a phantom model with and without SR reconstruction. Our results suggest that intravascular hemodynamic parameters might offer more robust measures of the flow field. The implementation of the SR model outside of the training space requires careful examination and generalizability necessitates further evidence.

The geometrical processing yielded mean (± SD) surface distance of 0.31 mm (± 0.06 mm), approximately 38% (± 7.5%) of the native voxel size. The resulting Hausdorff distance was 1.2 mm or 142% of the voxel size. The region with highest error in the ECA branch (Fig. 3b) has 2–4 voxels across the luminal diameter, which is smaller than the previously reported limit of 4 voxel [28] or 8 voxel [29] for a bias < 5%. Previously, a study on vessel lumen segmentation of carotid subjects acquired in a 3 Tesla scanner reported mean Hausdorff distance of 0.44 mm or 73% of voxel size between machine- and human-segmented results on healthy subjects [30]. Although those data were acquired using a T1-weighted black-blood sequence, which is known to offer superior contrast for vessel wall imaging. Similarly, segmentations performed on co-registered Time-of-Flight sequence also help reduce segmentation errors [20]. In another study with 58 carotid subjects imaged with computed tomography angiography [31], the 95 percentile Hausdorff distance between the machine- and human-segmented luminal geometry was reported to be 8.95 ± 8.60 mm or up to 20 times of the voxel size ($0.45\times 0.45\times 0.64\text{ m}{\mathrm{m}}^{3}$). The uncertainty of geometrical processing reported in this study falls close to the lower limit of the uncertainty range across modalities, though deviations in stagnant velocity regions such as that in the carotid bulb, where magnitude image intensity is low, may persist (Figs. 3 and 4). Ideally, it is technically feasible to perform segmentation on the super-resolved velocity field by setting a velocity magnitude threshold at zero. However, the results were highly sensitive to the threshold and the background noise level in our preliminary analysis, suggesting a poor generalizability of this approach. To improve the status quo, SR models with geometry outputs will be highly desirable.

Regarding the velocity field, several studies have demonstrated improvements over native 4D-flow MRI velocities with the adoption of 4DFlowNet [17–19, 32]. In our study, the patterns of velocity contours demonstrated significant improvements over the native 3T results (Fig. 4a) in agreement with previous observations. The smoothness of the velocity magnitude and the relative error are visually improved (Fig. 4a, b). Such improvements are most obvious in recirculation regions in the carotid bulb (Fig. 4b, middle row) and the ICA branch (Fig. 4b, top two rows). Velocity profiles extracted across the center of mass (Fig. 4c) revealed larger discrepancies close to the boundaries, where the velocity gradient is typically underestimated. This can be attributed to the low-pass filtering effect associated with the resolution and the ambiguity in vessel boundary pixels, which poses a challenge to the subsequent calculation of the WSS.

For quantitative comparisons, a previous study showed RE of 30% in velocity magnitude between native and super-resolved data in cerebrovascular flow [19]. However, these metrics were calculated between the SR velocity field and the down-sampled CFD data onto the SR spatial grid, which is already biased from the native CFD space caused by the low-pass filtering. In this study, we avoided resampling of the velocity field and presented statistical measures on native CFD results directly with (non-)robust statistics of velocity and vorticity magnitudes (Table 2). The RE in this case can be larger than previously reported values but should present the statistical differences more truthfully. The maximum RE in the 3T SR case was observed for the skewness of velocity magnitude in the CCA (104%), yet still an 80% improvement over the 3T case. It is noteworthy that on the same CCA plane (*z* = 52.4 mm, Table 2), the RE for mean and skewness velocity was 0.07 and 1.04, respectively, suggesting the differential power of higher order statistics. Nevertheless, the non-parametric paired comparison showed that the RE for 3T SR was significantly smaller than the 3T case (*p* = 0.033), which supports the adoption of intravascular parameters along with their higher order statistics as hemodynamic markers.

Since imaging-derived flow rates are often used to set up the boundary conditions of digital twins, its uncertainty was also investigated in this study. The SD of Q from SR result (2.8%) was much better than the native 3T results (14.7%), especially in the ICA/ECA branches (Fig. 5). The mean *Q*, however, yielded 11.1% mean absolute error in the 3T SR case, higher than the 6.8% in the 3T case. This is partly attributed to the fact that velocities in the recirculation regions are often overestimated in the 3T case but corrected into lower velocities after the SR reconstruction (top two rows in Fig. 4a). This is also evident in Fig. 4c, where the SR results typically have lower peak velocities in the ECA and ICA. To compare, flow rates measured in a previous study by 4D-flow MRI yielded RE of 11–44% in a small-lumen vessel phantom with 2.2 mm inner diameter and isotropic voxel size of 2.5 to 7.5 mm [33]. In another study with 3 Tesla 4D-flow MRI and CFD, deviation in Q was found between 6.5–58% in the ICA of a phantom model [34]. In a vessel model with large internal diameter (22 mm) and Reynolds number up to 3500, the mean (± SD) error was up to 19% (± 17%) with 1 mm isotropic resolution in the non-stenotic part of the tube, and smaller voxel size did not always produce better results [35]. Our reported uncertainties are close to the lower limit of previously reported values. The SR-derived flow rates in the CCA are better than the native 3T case. These results seem to suggest that the SR model may amplify the error if the variable is already biased in the original data, such as the flow rates in the ICA/ECA.

In the context of WSS, it has been considered an important hemodynamic feature for predicting adverse events in CA patients with two underlying rationales. First, excessive WSS exerted on the plaque surface may exceed a critical stress level and lead to plaque rupture [36–39]. Second, lower or oscillating WSS may promote plaque progression [11, 40, 41]. A mechanistic consensus remains elusive, and the evidence level remains low.

WSS quantification is highly sensitive to the imaging resolution [34], surface reconstruction methods [42], and the near-wall velocity field [27]. While a 15% deviation from theoretical values was shown in a simple straight tube phantom [43], severe stenotic cases demonstrated discrepancies of averaged WSS up to 38% [22]. In this study, the 3T SR results demonstrated better estimation of the maximum, SD, and 97.5 quantile of WSS, but no statistical difference was found in these parameters against the 3T case (*p* = 0.2), even though visual interpretation suggested a better match to the CFD result (Fig. 6). At least part of the discrepancies can be explained by the absence of a SR geometry, as discussed above, which could potentially offer a better approximation of the true geometry. Higher resolution in the native 4D-flow MRI data could potentially solve the problem but will prolong the scan time in a way that it is not feasible for patients anymore.

Further, the comparisons of WSS are made directly with the CFD result without resampling, unlike many previous studies where CFD results would be down sampled onto 3T and 3T SR grids. Such resampling does offer better comparisons (see Figure S1 in the Supplementary Material) due to the low pass filtering effect; the true uncertainty is, however, inevitably underestimated, offering limited translational value for the results.

The results from this study demonstrated the benefits and limitations of the 4DFlowNet in super-resolving 4D-flow MRI-derived hemodynamic parameters at 3 Tesla. Improvements were found in qualitative assessments of flow patterns, intravascular parameters, and peak WSS. The discussion also underscores the importance of domain generalization in supervised learning. Although the recent ensemble learning strategy showed preferred overall performance, the improvements in domain-specific data were limited [19]. In this regard, a SR model for CA trained on a larger domain-specific cohort might offer better translational value. Further, without considering the geometry in the SR model, measurements relying on the near-wall flow are likely to suffer from higher levels of uncertainties than those reported in this study when pathologies are present. A sub-routine for geometry integrated into the SR pipeline will likely offer better results.

## Limitations

This study presents an exploratory investigation on the uncertainties of 4D-flow MRI measurements in a carotid model with and without 4DFlowNet enhancement, compared against its digital twin established with CFD. Several limitations in this study are acknowledged.

First, the in vitro phantom was derived from one healthy carotid subject. Although recirculation zones were present, the flow was steady and transient effects caused by pulsatility were not modeled. A larger real-world dataset with more patient-specific morphological and pathological conditions should be explored to test the findings presented in this study. Second, the pre-trained SR network was implemented as is. A domain-specific training exercise with a larger sample size and more physio-pathological conditions may yield better results. Additionally, we briefly tested the idea of simplifying the input of the network using just the magnitude image for the anatomical path, and comparisons of velocity magnitude are presented in the Supplementary Material (Figure S2). It seems feasible to simplify the network architecture in future works. Finally, comparisons made between 4D-flow MRI and CFD results are widely accepted yet remain arguable because neither of them can be regarded as clinical ground truth without further justification. Ideally, subsequent models should be calibrated on clinically relevant endpoints to further evaluate the translational value of SR models in 4D-flow MRI-derived hemodynamic analysis.

Despite its limitations, this study was conducted in a clinically relevant setting using a clinical 4D-flow MRI sequence. The reported findings should be therefore interpreted as a clinical best-case scenario. Further investigations are warranted to examine the diagnostic power of 4D-flow MRI data with SR models.

## Supplementary Information

Below is the link to the electronic supplementary material.

ESM 1 (DOCX 724 KB)

## Data Availability

The carotid model used in this study can be freely downloaded from https://grabcad.com/library/carotid-bifurcation-with-joined-outlets-1. Other data may be made available to qualified researchers upon reasonable request.

## Code Availability

The model and pretrained weights were downloaded from github.com/EdwardFerdian/4DFlowNet.
