# Supplementary material for: Uncertainty Quantification in Hemodynamic Metrics from 4D Flow MRI with Super-resolution in a Carotid Bifurcation Model
Source: J Imaging Inform Med. 2026 Jan 7;39(5):4212–22. doi: 10.1007/s10278-025-01796-w (PMC13694586; doi:10.1007/s10278-025-01796-w)
Supplement: Supplementary file 1 [file 10278_2025_1796_MOESM1_ESM.docx]

**Supplementary material**

**Uncertainty quantification in hemodynamic metrics from 4D flow MRI with super-resolution in a carotid bifurcation model**

Figure S1 WSS contours of (a) 3T measurements (top) and CFD-derived WSS sampled on to 3T grid (bottom). (b) 3T SR results (top) and CFD-derived WSS sampled on to 3T SR grid (bottom).

Figure S2 Comparisons between two input methods, with magnitude image only (Mag) and with magnitude and speed images (Mag + Speed) for (a) contours of differences in velocity magnitude and (b) velocity profiles across center of mass, corresponding to Figure 4c in the main manuscript. Two observations are worth noticing. First, the differences in velocity magnitude appears better in the CCA for the Mag method. Second, in high velocity regions, noticeably in the ICA and ECA, peak velocities are actually slightly underestimated for the Mag + Speed method, although the overall relative error is smaller. This probably suggests that using just the magnitude image to update the 4DFlowNet architecture poses a viable idea. Such claims are subject to further validations.

Table S1 Further statistical measures at different z locations for 3T and 3T SR cases against CFD, along with the relative error for each variable.

| Z [mm] | Var | CFD | 3T | 3T SR | RE (3T) | RE (3T SR) |
| --- | --- | --- | --- | --- | --- | --- |
| 73.25 | Kurtosis Velocity | -0.685 | -0.878 | -1.215 | 0.282 | 0.774 |
| 73.25 | Kurtosis Vorticity | -0.519 | 1.918 | -0.452 | 4.697 | 0.129 |
| 73.25 | Mean Velocity | 0.062 | 0.065 | 0.057 | 0.052 | 0.084 |
| 73.25 | Mean Vorticity | 0.083 | 0.044 | 0.046 | 0.465 | 0.448 |
| 73.25 | SD Velocity | 0.031 | 0.033 | 0.032 | 0.055 | 0.027 |
| 73.25 | SD Vorticity | 0.050 | 0.021 | 0.027 | 0.578 | 0.459 |
| 73.25 | Skewness Velocity | -0.097 | -0.160 | -0.194 | 0.648 | 0.999 |
| 73.25 | Skewness Vorticity | 0.557 | 1.055 | 0.561 | 0.894 | 0.007 |
| 70.94 | Kurtosis Velocity | -0.524 | -0.744 | -1.201 | 0.420 | 1.292 |
| 70.94 | Kurtosis Vorticity | -0.888 | 0.127 | -0.175 | 1.143 | 0.803 |
| 70.94 | Mean Velocity | 0.060 | 0.061 | 0.055 | 0.011 | 0.094 |
| 70.94 | Mean Vorticity | 0.084 | 0.042 | 0.042 | 0.497 | 0.492 |
| 70.94 | SD Velocity | 0.030 | 0.031 | 0.031 | 0.035 | 0.032 |
| 70.94 | SD Vorticity | 0.052 | 0.017 | 0.026 | 0.664 | 0.506 |
| 70.94 | Skewness Velocity | 0.111 | -0.070 | -0.179 | 1.628 | 2.613 |
| 70.94 | Skewness Vorticity | 0.470 | 0.730 | 0.755 | 0.555 | 0.607 |
| 68.62 | Kurtosis Velocity | -0.501 | -0.285 | -0.941 | 0.431 | 0.877 |
| 68.62 | Kurtosis Vorticity | 0.053 | 2.412 | 0.771 | 44.369 | 13.498 |
| 68.62 | Mean Velocity | 0.053 | 0.056 | 0.049 | 0.067 | 0.077 |
| 68.62 | Mean Vorticity | 0.075 | 0.036 | 0.036 | 0.516 | 0.516 |
| 68.62 | SD Velocity | 0.032 | 0.030 | 0.030 | 0.043 | 0.051 |
| 68.62 | SD Vorticity | 0.051 | 0.017 | 0.023 | 0.669 | 0.545 |
| 68.62 | Skewness Velocity | 0.591 | 0.413 | 0.194 | 0.302 | 0.671 |
| 68.62 | Skewness Vorticity | 0.898 | 1.419 | 1.188 | 0.580 | 0.324 |
| 66.3 | Kurtosis Velocity | -0.476 | 0.032 | -0.629 | 1.068 | 0.322 |
| 66.3 | Kurtosis Vorticity | 2.027 | 2.836 | 1.867 | 0.399 | 0.079 |
| 66.3 | Mean Velocity | 0.044 | 0.048 | 0.040 | 0.092 | 0.097 |
| 66.3 | Mean Vorticity | 0.064 | 0.030 | 0.031 | 0.531 | 0.512 |
| 66.3 | SD Velocity | 0.034 | 0.031 | 0.031 | 0.105 | 0.103 |
| 66.3 | SD Vorticity | 0.055 | 0.018 | 0.025 | 0.679 | 0.553 |
| 66.3 | Skewness Velocity | 0.794 | 0.798 | 0.672 | 0.006 | 0.154 |
| 66.3 | Skewness Vorticity | 1.544 | 1.650 | 1.548 | 0.069 | 0.003 |
| 63.99 | Kurtosis Velocity | -0.506 | 0.277 | -0.451 | 1.546 | 0.111 |
| 63.99 | Kurtosis Vorticity | 3.138 | 3.152 | 1.354 | 0.004 | 0.569 |
| 63.99 | Mean Velocity | 0.039 | 0.042 | 0.034 | 0.077 | 0.125 |
| 63.99 | Mean Vorticity | 0.058 | 0.031 | 0.031 | 0.469 | 0.459 |
| 63.99 | SD Velocity | 0.036 | 0.033 | 0.034 | 0.081 | 0.055 |
| 63.99 | SD Vorticity | 0.059 | 0.022 | 0.028 | 0.633 | 0.532 |
| 63.99 | Skewness Velocity | 0.866 | 0.997 | 0.851 | 0.151 | 0.017 |
| 63.99 | Skewness Vorticity | 1.828 | 1.705 | 1.326 | 0.067 | 0.274 |
| 61.67 | Kurtosis Velocity | -0.675 | -0.735 | -0.782 | 0.088 | 0.158 |
| 61.67 | Kurtosis Vorticity | 6.670 | 1.515 | 1.240 | 0.773 | 0.814 |
| 61.67 | Mean Velocity | 0.038 | 0.044 | 0.034 | 0.159 | 0.110 |
| 61.67 | Mean Vorticity | 0.057 | 0.033 | 0.031 | 0.423 | 0.450 |
| 61.67 | SD Velocity | 0.037 | 0.034 | 0.036 | 0.077 | 0.036 |
| 61.67 | SD Vorticity | 0.074 | 0.022 | 0.031 | 0.699 | 0.586 |
| 61.67 | Skewness Velocity | 0.834 | 0.639 | 0.772 | 0.234 | 0.075 |
| 61.67 | Skewness Vorticity | 2.550 | 1.225 | 1.264 | 0.520 | 0.504 |
| 59.35 | Kurtosis Velocity | -1.220 | -1.022 | -1.037 | 0.162 | 0.150 |
| 59.35 | Kurtosis Vorticity | 23.747 | -0.807 | 0.335 | 1.034 | 0.986 |
| 59.35 | Mean Velocity | 0.035 | 0.042 | 0.032 | 0.198 | 0.085 |
| 59.35 | Mean Vorticity | 0.042 | 0.033 | 0.031 | 0.224 | 0.266 |
| 59.35 | SD Velocity | 0.031 | 0.030 | 0.034 | 0.060 | 0.083 |
| 59.35 | SD Vorticity | 0.051 | 0.019 | 0.029 | 0.622 | 0.431 |
| 59.35 | Skewness Velocity | 0.537 | 0.498 | 0.701 | 0.072 | 0.305 |
| 59.35 | Skewness Vorticity | 3.869 | 0.542 | 0.967 | 0.860 | 0.750 |
| 57.04 | Kurtosis Velocity | -0.549 | -0.592 | -0.885 | 0.078 | 0.612 |
| 57.04 | Kurtosis Vorticity | -0.284 | -0.700 | -0.251 | 1.463 | 0.118 |
| 57.04 | Mean Velocity | 0.031 | 0.040 | 0.033 | 0.282 | 0.084 |
| 57.04 | Mean Vorticity | 0.030 | 0.029 | 0.026 | 0.041 | 0.133 |
| 57.04 | SD Velocity | 0.034 | 0.034 | 0.040 | 0.006 | 0.173 |
| 57.04 | SD Vorticity | 0.025 | 0.016 | 0.027 | 0.367 | 0.055 |
| 57.04 | Skewness Velocity | 0.940 | 0.797 | 0.831 | 0.152 | 0.116 |
| 57.04 | Skewness Vorticity | 0.730 | 0.444 | 0.949 | 0.392 | 0.301 |
| 54.72 | Kurtosis Velocity | -1.262 | -1.186 | -1.459 | 0.060 | 0.157 |
| 54.72 | Kurtosis Vorticity | -0.739 | -1.074 | -0.593 | 0.454 | 0.197 |
| 54.72 | Mean Velocity | 0.042 | 0.052 | 0.050 | 0.236 | 0.178 |
| 54.72 | Mean Vorticity | 0.039 | 0.031 | 0.035 | 0.190 | 0.093 |
| 54.72 | SD Velocity | 0.038 | 0.038 | 0.042 | 0.007 | 0.116 |
| 54.72 | SD Vorticity | 0.026 | 0.016 | 0.026 | 0.405 | 0.015 |
| 54.72 | Skewness Velocity | 0.446 | 0.308 | 0.288 | 0.308 | 0.354 |
| 54.72 | Skewness Vorticity | 0.435 | 0.239 | 0.712 | 0.451 | 0.636 |
| 52.4 | Kurtosis Velocity | -1.118 | -1.224 | -1.439 | 0.094 | 0.286 |
| 52.4 | Kurtosis Vorticity | -0.524 | -0.729 | -0.703 | 0.392 | 0.343 |
| 52.4 | Mean Velocity | 0.063 | 0.061 | 0.059 | 0.041 | 0.067 |
| 52.4 | Mean Vorticity | 0.058 | 0.036 | 0.039 | 0.383 | 0.318 |
| 52.4 | SD Velocity | 0.034 | 0.036 | 0.040 | 0.052 | 0.180 |
| 52.4 | SD Vorticity | 0.034 | 0.017 | 0.024 | 0.497 | 0.271 |
| 52.4 | Skewness Velocity | -0.169 | 0.147 | 0.007 | 1.871 | 1.041 |
| 52.4 | Skewness Vorticity | 0.596 | 0.330 | 0.648 | 0.447 | 0.086 |
| 50.09 | Kurtosis Velocity | -1.101 | -1.187 | -1.426 | 0.078 | 0.295 |
| 50.09 | Kurtosis Vorticity | 0.870 | -1.114 | -0.025 | 2.281 | 1.029 |
| 50.09 | Mean Velocity | 0.063 | 0.059 | 0.055 | 0.072 | 0.135 |
| 50.09 | Mean Vorticity | 0.057 | 0.033 | 0.036 | 0.411 | 0.358 |
| 50.09 | SD Velocity | 0.033 | 0.035 | 0.039 | 0.050 | 0.183 |
| 50.09 | SD Vorticity | 0.037 | 0.013 | 0.023 | 0.635 | 0.373 |
| 50.09 | Skewness Velocity | -0.142 | 0.140 | 0.072 | 1.989 | 1.507 |
| 50.09 | Skewness Vorticity | 1.146 | 0.089 | 0.811 | 0.923 | 0.292 |
| 47.77 | Kurtosis Velocity | -1.136 | -1.222 | -1.454 | 0.076 | 0.280 |
| 47.77 | Kurtosis Vorticity | 0.135 | -0.905 | -0.061 | 7.712 | 1.454 |
| 47.77 | Mean Velocity | 0.060 | 0.052 | 0.048 | 0.131 | 0.207 |
| 47.77 | Mean Vorticity | 0.053 | 0.029 | 0.031 | 0.447 | 0.422 |
| 47.77 | SD Velocity | 0.033 | 0.035 | 0.037 | 0.060 | 0.147 |
| 47.77 | SD Vorticity | 0.030 | 0.012 | 0.022 | 0.600 | 0.273 |
| 47.77 | Skewness Velocity | -0.083 | 0.258 | 0.195 | 4.119 | 3.357 |
| 47.77 | Skewness Vorticity | 0.845 | 0.108 | 0.843 | 0.873 | 0.003 |
